# Validation of the Opening Minds Scale and patterns of stigma in Chilean primary health care

**DOI:** 10.1371/journal.pone.0221825

**Published:** 2019-09-05

**Authors:** Jaime C. Sapag, Rachel Klabunde, Luis Villarroel, Paola R. Velasco, Cinthia Álvarez, Claudia Parra, Sireesha J. Bobbili, Franco Mascayano, Inés Bustamante, Rubén Alvarado, Patrick Corrigan

**Affiliations:** 1 Department of Public Health, School of Medicine, Faculty of Medicine, Pontificia Universidad Católica de Chile, Santiago, Chile; 2 Division of Clinical Public Health, Dalla Lana School of Public Health, University of Toronto, Ontario, Canada; 3 WHO/PAHO Collaborating Centre, Institute for Mental Health Policy Research, Centre for Addiction and Mental Health (CAMH), Ontario, Canada; 4 Department of Epidemiology, Mailman School of Public Health, Columbia University, New York City, New York, United States of America; 5 Division of Behavioral Health Services and Policy Research, New York State Psychiatric Institute, New York City, New York, United States of America; 6 Faculty of Public Health and Administration, Universidad Peruana Cayetano Heredia, Lima, Peru; 7 Mental Health Program, School of Public Health, Faculty of Medicine, Universidad de Chile, Santiago, Chile; 8 Institute of Health Sciences, Universidad O’Higgins, Rancagua, Chile; 9 Department of Psychology, Lewis College of Human Sciences, Illinois Institute of Technology, Chicago, Illinois, United States of America; Universidad ESAN, PERU

## Abstract

**Objectives:**

Stigma toward people with mental health problems (MHP) in primary health care (PHC) settings is an important public health challenge. Research on stigma toward MHP is relatively scarce in Chile and Latin America, as are instruments to measure stigma that are validated for use there. The present study aims to validate the Opening Minds Scale for Health Care Professionals (OMS-HC) among staff and providers in public Chilean PHC clinics, and examine differences in stigma by sociodemographic characteristics.

**Methods:**

803 participants from 34 PHC clinics answered a self-administered questionnaire. Confirmatory factor analysis was completed. Average 15-item OMS-HC scores were calculated, and means were compared via t-test or ANOVA to identify group differences. Correlations of OMS-HC scores with other commonly used stigma scores were calculated to evaluate construct validity.

**Results:**

The 3-factor OMS-HC structure was confirmed in this population. The average OMS-HC (α = 0.69) score was 34.55 (theoretical range 15–75). Significantly lower (less stigmatizing) mean OMS-HC scores were found in those with additional training and/or personal experience with MHP.

**Conclusion:**

The validated, Spanish version of OMS-HC can be of use to further research stigma toward MHP in Chile and Latin America, advancing awareness and inspiring interventions to reduce stigma in the future.

## Introduction

Stigma toward people with mental health problems (MHP) is a significant public health challenge [1,2]. Stigma is a phenomenon comprising negative thoughts and actions toward a certain individual or group, and according to Link & Phelan, “exists when elements of labeling, stereotyping, separation, status loss, and discrimination occur together in a power situation that allows them”([3], p. 377). There is ample evidence of stigma about people with MHP in health care settings and by health care professionals [4–6]. Stigmatization is a barrier to adequate care for those with MHP [7,8], and it can lead to them receiving lower-quality physical health care services than others [9,10]. Experiencing stigma in health care can also result in reduced MHP treatment adherence and engagement [11] and a decrease in quality of life [12,13] for those with MHP.

Primary health care (PHC) provides an opportunity to strengthen care for MHP. The high prevalence of mental disorders among PHC patients [14] and the fact that most patients with MHP will access the health care system through PHC providers [15,16] make this an ideal setting to implement early screening and treatment strategies for MHP. Additionally, services to treat MHP at the PHC level could potentially reduce stigma and discrimination, as people with these conditions are generally treated by the same providers and in the same, community-based location as people with other health conditions [17]. However, studies in multiple locations have confirmed the presence of stigma in PHC settings [4,18,19].

In Latin America, mental health care services are increasingly being integrated into the PHC system [20], and within the region, Chile has been a leader of this model, with interdisciplinary family health teams in public PHC centers providing mental health services [21]. The system of public PHC centers (*Centros de Salud Familiar*, CESFAM), as well as other public mental health centers, specialty care establishments, and hospitals, serves more than 75% of the Chilean population (>13.6 million people) [22,23]. Residents covered by public insurance register at the CESFAM that is located closest to their home and receive PHC services there, including the mental health services mentioned above [22].

There is limited research on stigma toward MHP in PHC settings in this region [24,25], despite the significant treatment gap for MHP and the reported stigmatizing attitudes among providers [25,26]. In Chile, specifically, only 38.5% of patients with a psychiatric diagnosis receive any kind of mental health care treatment [19]. One challenge for understanding and addressing stigma toward MHP in Chile is the lack of validated, culturally relevant instruments to measure the phenomenon [27].

To address this gap and enable further study of stigma in this context, the present study seeks to examine the results of the cross-culturally adapted Spanish Opening Minds Stigma Scale for Health Care Providers (OMS-HC) in Chilean PHC settings, exploring differences in levels of stigma by sociodemographic groups, and validate the instrument through analysis of its internal consistency, factor structure, and correlation with other scales commonly used to measure stigma.

## Materials and methods

The current study is part of a cross-sectional project designed to investigate the presence of stigma toward MHP and substance use issues in Chilean PHC (“Stigma toward Mental Illness in Primary Health Care Professionals in Chile”, FONDECYT Regular #1160099).

### Measures

PHC staff and providers completed the self-administered questionnaire in Spanish, which included a battery of scales to measure various aspects of stigma toward MHP and substance use issues. The OMS-HC was developed in Canada to measure attitudes and behavioral intentions toward MHP in health care providers, and one of the highest-quality instruments to measure stigma [28]. Created with a total of 20 items, initial factor analysis resulted in a two-factor, 12-item version of the scale [28]. However, a subsequent validation study resulted in a three-factor, 15-item version [29], which has been used in Canada [30] as well as internationally, including in Italy [31], Pakistan [32], and Singapore [33]. This version of the OMS-HC was used in the present study. The OMS-HC was chosen for this study because of its strong psychometric properties from prior validations [29–30], particularly in the PHC setting [30], and wide international use [31–33], as well as because research team members had been working on stigma research using this instrument since 2009. The cross-culturally adapted, 15-item OMS-HC score was analyzed as the primary outcome of interest.

A multi-phase cross-cultural adaptation process was completed, including nine phases (preparation; three independent translations; expert committee synthesis; researcher and PHC professional focus groups and PHC user cognitive interviews; expert committee synthesis 2; two independent back translations; expert committee synthesis 3; pilot testing; and final revisions). These phases were developed based on recommendations from Beaton et al. [34] and the WHO [35] and incorporated an array of diverse voices from PHC contexts in Chile to meet three Adaptation Objectives defined prior to beginning the adaptation process (Understandability, Relevance, and Acceptability and Answer Options) [35]. The original, English version and the cross-culturally adapted Spanish version can be found as Supplemental Information for this study (see S1 and S2 Files).

Multiple international validations of the OMS-HC [29–31], have found a three-factor structure of the 15-item scale, and we sought to examine this structure in the present study to facilitate international comparison. This instrument can result in a possible score of 15 to 75, with a higher score indicating more stigmatizing attitudes and behavioral intentions [28]. Questions in the scale address attitudes regarding disclosure of MHP, attitudes toward individuals with MHP, and the social distance professionals would like to have with people who have MHP. Response options are presented in a 5-item Likert scale (Strongly Disagree = 1, Disagree = 2, Neither Agree nor Disagree = 3, Agree = 4, and Strongly Agree = 5). Seven items in the scale require reverse coding [29].

The questionnaire also included cross-culturally adapted versions of other scales used to measure different aspects of stigma toward MHP or substance use issues. The Mental Illness: Clinician’s Attitudes (MICA) scale was designed to measure attitudes toward people with MHP in students and health professionals, and contains 16 items, with a theoretical score range form 16 (least stigmatizing) to 96 (most stigmatizing) [36]. The Modified Bogardus Social Distance (MBSD) scale measures attitudes toward certain populations by focusing on the types of relationships the respondent would be willing to have with members of that population [37]; in this case, two versions of the scale were used, referring to people with schizophrenia and cocaine paste dependence. The scale has a theoretical range of 6 to 24, with higher scores indicating greater social distance. The Recovery Scale (RS) measures beliefs regarding the potential of people with severe mental illness to recover (in this case, a severe mental illness or a severe addiction to a psychoactive substance) [38]. The RS has 13-items, its theoretical range is from 13 to 117, with higher scores demonstrating negative views about recovery potential. Finally, the Attribution Questionnaire (AQ-27) evaluates nine stereotypes about people with mental illness (in this case, schizophrenia), with a range of 3 to 27 to for each stereotype and higher scores indicating greater endorsement of the stereotype [38].

Demographic information was also collected from PHC staff and providers, including variables such as age, gender identity, marital status, nationality, indigenous identity, religious beliefs, role within the CESFAM, experience working in PHC, educational background, among other. There were also questions specifically regarding the participant’s experience with mental health care in the CESFAM, how important they believed mental health/substance use services are within the CESFAM, and personal experience (whether in themselves or close family/friends) with MHP, as these variables have been related to stigma among health care providers in the literature [32,39–41].

### Sample

To sample CESFAMs, the research team first contacted all public Health Districts in the country. Out of 29 Health Districts, 14 expressed interest in participating in the study and eight were selected, representing seven out of 16 regions in Chile, from Atacama in the North to the Lakes region in the South. Municipalities within participating health districts and Directors of each CESFAM were contacted to receive approval.

Once CESFAM participation was confirmed, data collection was scheduled between March and October 2017, with special meeting times set aside by CESFAM Directors to encourage participation. Approximately 50% of providers and staff responded to the survey in these meeting times. All PHC staff and providers attending these meetings filled out the self-administered questionnaires after providing informed consent. In the Coquimbo region, because of a scheduling conflict, none of the physicians from the five CESFAMs there were able to participate, so the research team scheduled a second data collection date there, with successful participation of physicians at that time. Inclusion criteria for PHC staff included being an active, hired, and full-time member of the CESFAM staff at the time of data collection.

### Analysis

The present study analyzed the sample of PHC staff and providers that responded to all OMS-HC items. To assess the factor structure suggested by Modgill et al. for the 15-item OMS-HC scale [29], confirmatory factor analysis was performed using structural equation modeling (SEM). The Chi-squared test was used to analyze the discrepancy between data and the theoretical model implied; although this test is sensitive to sample size, in this case it is valid, as the sample size was large (n = 803). To evaluate model fit, the root mean square error of approximation, comparative fit index, Tucker-Lewis Index, standardized root mean square residual, and coefficient of determination were calculated. Internal consistency of the OMS-HC scale was evaluated using Chronbach’s α. To evaluate construct validity of the scales, Pearson correlations were calculated between the OMS-HC scores and other stigma scale scores (MICA, MBSD, RS, AQ-27). Individual and average OMS-HC scale scores were computed, and mean scores between sociodemographic groups were compared using Student’s t-test and ANOVA with Bonferroni adjustment for multiple comparisons. Findings from these comparisons were confirmed using non-parametric tests (Wilcoxon ranks and Kruskal-Wallis). Statistical analysis was performed using Stata 14 and SPSS Version 17.

This study was approved by the Research Ethics Committee at the Pontificia Universidad Católica de Chile, approval number 16–090.

## Results

Sociodemographic characteristics of CESFAM staff and providers are provided in Table 1.

**Table 1:** Sociodemographic characteristics of sample of Chilean primary health care staff and providers (N = 803).

Characteristic	N (%)
Gender	
Men	183 (23.2)
Women	607 (76.8)
Age (years)	
18–24	32 (4.1)
25–34	328 (41.9)
35–44	218 (27.9)
45–54	133 (17.0)
55+	71 (9.1)
Nationality	
Chilean	777 (97.9)
Other Nationality	17 (2.1)
CESFAM Area	
Health Team	603 (76.9)
Administrative	136 (17.3)
Maintenance/Security	9 (1.1)
Other	37 (4.7)
Additional Training in Mental Health	
Yes	233 (29.7)
No	552 (70.3)
Family Member with MHP	
Yes	342 (43.7)
No	421 (53.8)
Prefer Not to Answer	20 (2.5)
Friend with MHP	
Yes	313 (40.7)
No	436 (56.8)
Prefer Not to Answer	19 (2.5)
Personal Experience with MHP	
Yes	170 (22.0)
No	591 (76.3)
Prefer Not to Answer	13 (1.7)
Participant Role in CESFAM	
Early Childhood Educator	12 (1.6)
Nurse	70 (9.1)
Kinesiologist	50 (6.5)
Physician	60 (7.8)
Nutritionist	34 (4.4)
Midwife	50 (6.5)
Dentist	37 (4.8)
Administrative Personnel	104 (13.5)
Cleaning Personnel	7 (0.9)
Security Personnel	1 (0.1)
Psychologist	48 (6.2)
Pharmacist	6 (0.8)
Pharmaceutical Technician	14 (1.8)
Nursing Technician	152 (19.7)
Medical Technician	2 (0.3)
Occupational Therapist	8 (1.1)
Social Worker	50 (6.5)
Other	65 (8.4)

CESFAM = *Centro de Salud Familiar* (Chilean Public Primary Health Care Clinic), MHP = mental health problem.

A total of 803 professionals responded to all OMS-HC items and comprised the sample for this analysis, with a response rate of nearly 100%. The sample comprised mostly women (76.4%), the mean age of CESFAM staff and providers was 37.9 years, and nearly all participants were Chilean. A total of 19 different roles in the CESFAM, from cleaning personnel to nurses to physicians, were represented among survey respondents; more than 75% of participants belong to the health teams in the centers. On average, respondents have 10 years of experience working in PHC. Approximately 40% of the surveyed population had personal experience with MHP or substance use issues in either a close family member or friend, while 22% reported receiving treatment for these problems themselves.

Confirmatory factor analysis was performed to test whether the factor structure of the three subscales was appropriate in this sample. Item loadings from this analysis, as well as factor covariances, are presented in Table 2.

**Table 2:** Item loadings from confirmatory factor analysis in Opening Minds Scale for Health Care Providers (OMS-HC).

Item No. and Text	Standardized Coefficient
Factor 1: Attitudes toward people with MHP	
1: I am more comfortable helping a person who has a physical illness than I am helping a person who has a mental illness.	0.347
12: Despite my professional beliefs, I have negative reactions towards people who have mental illness.	0.407
13: There is little I can do to help people with mental illness.	0.472
14: More than half of people with mental illness don’t try hard enough to get better.	0.523
18: Health care providers do not need to be advocates for people with mental illness.	0.306
20: I struggle to feel compassion for a person with mental illness.	0.321
Factor 2: Attitudes toward Disclosure of MHP	
4: If I were under treatment for a mental illness, I would not disclose this to any of my colleagues.	0.387
6: I would see myself as weak if I had a mental illness and could not fix it for myself.	0.565
7: I would be reluctant to seek help if I had a mental illness.	0.371
10r: If I had a mental illness, I would tell my friends.	0.424
Factor 3: Social Distance from those with MHP	
3r: If a colleague with whom I work told me they had a managed mental illness, I would be as willing to work with him/her.	0.385
8r: Employers should hire a person with a managed mental illness if he/she is the best person for the job.	0.502
9r: I would still go to a physician if I knew that the physician had been treated for a mental illness.	0.649
17: I would not want a person with a mental illness, even if it were appropriately managed, to work with children.	0.467
19r: I would not mind if a person with a mental illness lived next door to me.	0.411
Subscale Covariances	
Subscale 1—Subscale 2	0.715
Subscale 1—Subscale 3	0.565
Subscale 2—Subscale 3	0.453

MHP = mental health problems. Item numbers refer to original, 20-item OMS-HC scale. “r” refers to items that have been reverse coded for scoring. Text presented here is original, English text (participants answered the items in Spanish from the cross-culturally adapted version of the scale).

Multiple indicators of model fit were calculated for this analysis. The root mean square error of approximation was 0.052 (considered excellent if <0.06 [42,43]), the standardized root mean square residual was 0.048 (considered excellent fit if <0.08 [42,43]), and the coefficient of determination R^2^ was 89%, all indicating excellent model fit. The Tucker Lewis Index (0.798) and Comparative Fit Index (0.832) were lower than the ideal of ≥0.95 [42,43].

Analysis of the internal consistency of the complete, 15-item OMS-HC scale showed an acceptable, though slightly low, internal consistency with a Cronbach’s α of 0.69. The three subscales identified by Modgill et al. [29], which correspond to attitudes toward people with MHP, attitudes toward disclosure of MHP, and social distance from those with MHP, had lower Cronbach’s α in this sample (0.53, 0.48, and 0.60, respectively).

To analyze construct validity of the OMS-HC, Pearson correlations were calculated for OMS-HC scores and other scale scores, and results are presented in Table 3. The OMS-HC scale had relatively strong positive correlations with the MICA (0.683), AQ-27 (0.572), and MBSD schizophrenia scores (0.504). Weak negative correlations were found with the Recovery Scales (-0.101 for severe mental illness, and -0.109 for severe psychoactive substance addiction). All correlations were statistically significant.

**Table 3:** Pearson correlations between Opening Minds Scale for Health Care Providers (OMS-HC) and other scale scores.

Scale	Pearson Correlation Coefficient	p-value
MICA	0.683	<0.001
MBSD-Schizophrenia	0.504	<0.001
MBSD-Cocaine Paste	0.381	<0.001
RS-Severe Mental Illness	-0.101	0.006
RS-Severe Psychoactive Substance Addiction	-0.109	0.003
AQ-27	0.572	<0.001

MICA = Mental Illness: Clinician’s Attitudes, MBSD = Modified Bogardus Social Distance, RS = Recovery Scale, AQ-27 = Attribution Questionnaire. Correlation with the AQ-27 was performed with the total sum of all stereotype scores.

In the overall sample, the mean OMS-HC score was 34.55, with a range of 15 to 58 and a standard deviation of 7.02. Exploration and comparison of means by sociodemographic characteristics (see Table 4) revealed statistically significantly lower levels of stigma (lower OMS-HC score) for the Health Team compared with the Administrative team in the CESFAM. Additionally, those with additional training in mental health had a significantly lower average OMS-HC score than those without training. Similarly, CESFAM staff or professionals who had a family member, friend, or their own personal experience with MHP or substance use issues had lower average OMS-HC scores than those who did now know someone who has received treatment for these issues.

**Table 4:** Mean Opening Minds Scale for Health Care Providers (OMS-HC) scores by sociodemographic characteristics.

Characteristic	Mean OMS-HC Score	Standard Deviation	p-value
Gender			0.521
Male	34.27	7.30	
Female	34.65	6.94	
Age (years)			0.177
18–24	32.78	6.33	
25–34	34.45	6.57	
35–44	33.92	7.22	
45–54	35.26	7.63	
55+	35.78	7.49	
CESFAM Area			<0.001
Health Team	33.79	6.81	
Administrative	36.95	7.06	
Maintenance/Security	39.00	8.77	
Other	35.57	6.74	
Additional Training in Mental Health			<0.001
Yes	32.24	7.06	
No	35.46	6.74	
Family Member with MHP/Substance Use Issues			0.002
Yes	33.60	6.96	
No	35.18	6.96	
Friend with MHP/Substance Use Issues			<0.001
Yes	33.09	6.83	
No	35.26	6.78	
Personal Experience with MHP/Substance Use Issues			<0.001
Yes	32.74	7.13	
No	34.88	6.91	

CESFAM = *Centro de Salud Familiar* (Chilean Public Primary Health Care Clinic); MHP = mental health problems. Student T-Test and ANOVA used to compare means, findings confirmed with non-parametric Wilcoxon ranks test and Kruskal-Wallis tests.

**Fig 1:**
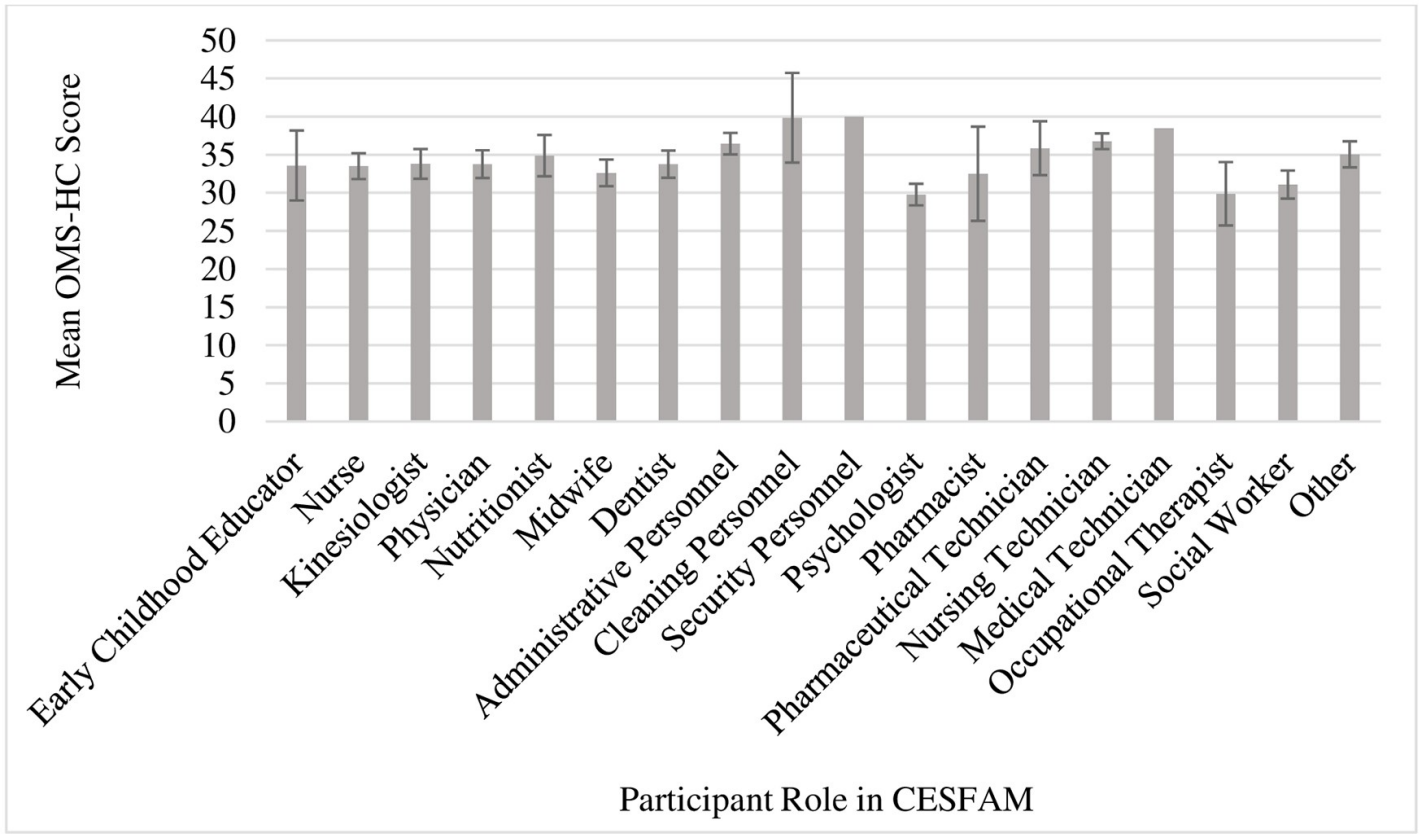
Mean Opening Minds Scale for Health Care Providers (OMS-HC) score by participant role in CESFAM. Note: CESFAM = *Centro de Salud Familiar* (Chilean Public Primary Health Care Clinic). Bars represent 95% Confidence Interval. Security Personnel and Nursing Technician do not have Confidence Intervals represented due to small sample size.

Finally, there were some differences in average OMS-HC score by CESFAM role, as demonstrated in Fig 1. Psychologists had the lowest mean score (29.77), followed by occupational therapists (29.88) and social workers (31.08). Security personnel, cleaning personnel, and medical technicians had the highest averages of the sample, with mean OMS-HC scores of 40.00, 39.86, and 38.50, respectively. No differences in OMS-HC score were observed between men and women or between age groups.

## Discussion

The main goal of this study was to validate and examine the factor structure of the cross-culturally adapted version of the OMS-HC in the Chilean context. Confirmatory factor analysis was completed, with multiple measures of model fit found to be acceptable and confirming the validity of the three-factor structure from Modgill et al [29] among Chilean PHC staff and providers. This three-factor structure was also validated in additional studies in Canada [30], with a similar three-factor structure containing 14 items found to be valid in Singapore [33]. In this validation, the Cronbach’s α for the three subscales were low, indicating that it is more appropriate, in the Chilean context, to utilize the complete, 15-item OMS-HC scale rather than individual subscales.

We sought to assess construct validity of the OMS-HC scale in the Chilean PHC context by measuring the extent to which the scale scores correlated with other, commonly used measurements of aspects of stigma. The strong positive correlation between the OMS-HC scale and the MICA scale was encouraging, indicating appropriate measurement of stigma toward MHP in PHC staff and providers. Moderate correlations with measures of social distance (MBSD) and stereotypes (AQ-27) toward those with schizophrenia suggests that some aspects of these phenomenon are being captured by the OMS-HC, however there are others that the scale does not address. The slight negative correlations with both versions of the RS were somewhat surprising, as a larger total RS score indicates more negative feelings regarding the potential for recovery. However, the OMS-HC does not explicitly address the potential for recovery in those with MHP, and thus these negative correlations are not of concern.

Additionally, the present study also sought to examine the results of the application of the OMS-HC in PHC centers throughout Chile, and identified the presence of stigma toward those with MHP among PHC staff and providers. The average OMS-HC score of 34.55 among the 803 study participants indicates that there is stigmatization of people with MHP in this setting. When considering average scores from other international studies of stigma in health care providers, this finding is fairly consistent. In a study of stigma in Canadian community health centres, the average score for the 15-item OMS-HC among providers was 30.38 [30], while the average 14-item OMS-HC score among varying health care providers in Singapore was 35.7 [33] (indicating greater stigma, as the summation of scores for 15 items would be higher than this). Due to the importance of culture and social context in shaping stigma toward MHP [27,44], as well as the different versions of OMS-HC used in these studies, cross-national comparisons are imprecise. However, we conclude that Chilean PHC workers and professionals have levels of stigma that are similar to those in other countries.

Finally, comparison of mean OMS-HC scores in sociodemographic groups revealed that people with greater experience or contact with people with lived experience of mental illness or substance abuse had lower levels of stigma. Those with personal experience of mental illness or substance abuse, whether in themselves, a close family member, or friend, as well as those with additional training in mental health had lower levels of stigma toward MHP and substance abuse. Similarly, in the CESFAM, psychologists and social workers, who are typically involved in the mental health programs provided in the PHC centers, demonstrated less-stigmatizing attitudes and behavioral intentions than their peers. This suggests that including training regarding stigma and interactions with those with MHP could be a viable strategy for intervention to reduce stigma, which is consistent with findings from intervention studies that recognize increased social contact with people in recovery and training regarding stigma as important elements for intervention success [45–47]. Targeting interventions toward those that do not typically interact with patients with MHP and substance use issues in the PHC setting may also be a valuable strategy for future work. In addition, the Spanish language OMS-HC could be used in healthcare settings in Chile and elsewhere to identify staff and providers that have greater stigma toward MHP and refer them to stigma reduction interventions or programs.

There are some limitations of this study. The self-report nature of the OMS-HC creates the potential for social desirability bias in the results, which could lead to artificially low OMS-HC scores. However, this may have been mitigated by the anonymous nature of the survey. Additionally, as this was a cross-sectional sampling of Chilean PHC professionals, differences between OMS-HC scores in subgroups of staff and providers should not be considered causal. Finally, the internal consistency found for the 15-item OMS-HC scale is low, just below the generally accepted α = 0.7, and some indicators of model fit in the confirmatory factor analysis were lower than ideal. It is possible that, in the Chilean context, the cross-culturally adapted version of the OMS-HC does not fully measure the concept of stigma, despite favorable pilot and cognitive testing during the adaptation process. Further research is required using this cross-culturally adapted scale to examine ways to improve reliability. Furthermore, future research incorporating the patient perspective and experience of stigma in PHC could add an important dimension to the understanding of stigma toward MHP in Chile, as PHC staff and providers may have bias when reporting their own ideas regarding MHP and stigma. While some research has been conducted to incorporate and measure the patient perspective on MHP stigma in Chile and the Latin America region [25,48], more should be done.

This, the present study represents the first large scale examination of stigma toward MHP in PHC providers in Chile, and is an important step forward for stigma studies in South America.

## Conclusion

This study contributes to knowledge regarding stigma toward MHP in Chile, and validates the OMS-HC for future use in stigma studies in Chile and Latin America as a whole. The stigma results presented here, showing the presence of stigma toward MHP in Chilean CESFAM, demonstrate the need for future interventions to address and reduce this stigma in the region. Additionally, future use of the cross-culturally adapted Spanish language OMS-HC will be important for regional comparisons and further study. To address stigma in this context, this research team has applied for funding for an intervention study in CESFAMs in Chile to seek to reduce stigma toward MHP in PHC. Additionally, in July 2018, the Consortium for Stigma Research in Mental Health and Substance Use Issues in the Americas was created by fourteen practitioners, policy makers, and researchers representing six countries in the region to advance local research and interventions to reduce stigma toward MHP.

## Supporting information

S1 File Opening Minds Survey Spanish. **Opening Minds Scale for Health Care Professionals (OMS-HC) in Spanish.** This is the cross-culturally adapted Spanish version of the Opening Minds Scale for Health Care Professionals (OMS-HC) used in the present study.
(PDF)

S2 File Opening Minds Survey English. **Opening Minds Scale for Health Care Professionals (OMC-HC) in English.** This is the original version of the Opening Minds Scale for Health Care Professionals (OMS-HC), which was cross-culturally adapted for use in the present study in Spanish (for Spanish version, see S1 File).
(PDF)
