# Supplementary material for: Validation of the Opening Minds Scale and patterns of stigma in Chilean primary health care
Source: PLoS One. 2019 Sep 5;14(9):e0221825. doi: 10.1371/journal.pone.0221825 (PMC6728029; doi:10.1371/journal.pone.0221825)
Supplement: S1 File — Opening Minds Scale for Health Care Professionals (OMS-HC) in Spanish. This is the cross-culturally adapted Spanish version of the Opening Minds Scale for Health Care Professionals (OMS-HC) used in the present study. (PDF) [file pone.0221825.s001.pdf]

PONTIFICIA UNIVERSIDAD CATÓLICA DE CHILE

UNIVERSIDAD DE CHILE

Estigma hacia la Enfermedad Mental entre Profesionales de la Atención Primaria en Chile

CUESTIONARIO PARA PERSONAL DE CESFAMs

A continuación, se presentan varias preguntas que indagan sobre sus pensamientos, creencias, sentimientos, actitudes, comportamientos y experiencias relacionadas a personas con problemas de salud mental y/o de consumo de sustancias psicoactivas (drogas). No hay respuestas correctas, ni incorrectas. Por favor, indique la alternativa de respuesta que mejor describa su experiencia.

El cuestionario consta de varios componentes, entre los que también se pregunta información general sobre usted. Incluye varios instrumentos que se aplican internacionalmente en este tema. Su aplicación es parte del Proyecto FONDECYT ·1160099, "Estigma hacia la Enfermedad Mental entre Profesionales de la Atención Primaria en Chile".

Toda la información que nos brinde será completamente confidencial. Integraremos su información con la de otros participantes, para conocer las diferentes experiencias de las personas que trabajan en Atención Primaria. **Su participación es totalmente voluntaria y anónima**. No habrá ningún problema para usted si decide no participar.

Si hay alguna pregunta que usted prefiera no responder, puede omitirla, pero para esta investigación son muy importantes todas sus respuestas.

Se espera que demore aproximadamente 45 minutos o menos en responder el cuestionario.

Si tiene alguna duda, pregunte al personal que se encuentra aplicando el cuestionario.

Antes de comenzar, le reiteramos que todas sus respuestas e información personal serán completamente confidenciales.

¡Muchas gracias por su participación!

PARTE I

CUESTIONARIO A.

Las siguientes preguntas le piden manifestar su acuerdo o desacuerdo con una serie de enunciados sobre enfermedad mental. No hay una respuesta correcta a cada pregunta. Por favor, encierre en un círculo el número de la respuesta que mejor represente su opinión.

		**1** Muy en desacuerdo	**2** En desacuerdo	**3** Ni de acuerdo, ni en desacuerdo	**4** De acuerdo	**5** Muy de acuerdo
1.	Me siento más cómodo/a cuando atiendo a una persona que tiene una enfermedad física, que cuando atiendo a una persona que tiene una enfermedad mental.	**1** Muy en desacuerdo	**2** En desacuerdo	**3** Ni de acuerdo, ni en desacuerdo	**4** De acuerdo	**5** Muy de acuerdo
2.	Si una persona con una enfermedad mental se queja de síntomas físicos (ej.: náuseas, dolor de espalda o dolor de cabeza), probablemente se los atribuiría a su problema de salud mental.	**1** Muy en desacuerdo	**2** En desacuerdo	**3** Ni de acuerdo, ni en desacuerdo	**4** De acuerdo	**5** Muy de acuerdo
3.	Si un compañero de trabajo me dijera que tiene un trastorno de salud mental compensado, yo estaría igualmente dispuesto a trabajar con él/ella.	**1** Muy en desacuerdo	**2** En desacuerdo	**3** Ni de acuerdo, ni en desacuerdo	**4** De acuerdo	**5** Muy de acuerdo
4.	Si yo estuviera en tratamiento por una enfermedad mental, <u>no</u> se lo contaría a ninguno/a de mis compañeros/as de trabajo.	**1** Muy en desacuerdo	**2** En desacuerdo	**3** Ni de acuerdo, ni en desacuerdo	**4** De acuerdo	**5** Muy de acuerdo
5.	Yo estaría más dispuesto/a a buscar ayuda por una enfermedad mental si el profesional que me atendiera <u>no</u> estuviera relacionado con mi lugar de trabajo.	**1** Muy en desacuerdo	**2** En desacuerdo	**3** Ni de acuerdo, ni en desacuerdo	**4** De acuerdo	**5** Muy de acuerdo
6.	Me consideraría una persona "débil" si tuviera una enfermedad mental y <u>no</u> pudiera resolverla por mí mismo/a.	**1** Muy en desacuerdo	**2** En desacuerdo	**3** Ni de acuerdo, ni en desacuerdo	**4** De acuerdo	**5** Muy de acuerdo

7.	Yo estaría poco dispuesto/a a buscar ayuda si tuviera una enfermedad mental.	**1** Muy en desacuerdo	**2** En desacuerdo	**3** Ni de acuerdo, ni en desacuerdo	**4** De acuerdo	**5** Muy de acuerdo
8.	Si una persona que tiene una enfermedad mental compensada es la más apta para un trabajo, los empleadores deberían contratarla/o.	**1** Muy en desacuerdo	**2** En desacuerdo	**3** Ni de acuerdo, ni en desacuerdo	**4** De acuerdo	**5** Muy de acuerdo
9.	Si yo supiera que un médico ha sido tratado por una enfermedad mental, aun así me atendería con él/ella.	**1** Muy en desacuerdo	**2** En desacuerdo	**3** Ni de acuerdo, ni en desacuerdo	**4** De acuerdo	**5** Muy de acuerdo
10.	Si yo tuviera una enfermedad mental, se lo contaría a mis amigos/as.	**1** Muy en desacuerdo	**2** En desacuerdo	**3** Ni de acuerdo, ni en desacuerdo	**4** De acuerdo	**5** Muy de acuerdo
11.	Es responsabilidad del personal de salud transmitir una visión esperanzadora a las personas con una enfermedad mental.	**1** Muy en desacuerdo	**2** En desacuerdo	**3** Ni de acuerdo, ni en desacuerdo	**4** De acuerdo	**5** Muy de acuerdo
12.	A pesar de mis principios profesionales, tengo reacciones negativas hacia las personas que tienen una enfermedad mental.	**1** Muy en desacuerdo	**2** En desacuerdo	**3** Ni de acuerdo, ni en desacuerdo	**4** De acuerdo	**5** Muy de acuerdo
13.	Es poco lo que puedo hacer para ayudar a las personas con una enfermedad mental.	**1** Muy en desacuerdo	**2** En desacuerdo	**3** Ni de acuerdo, ni en desacuerdo	**4** De acuerdo	**5** Muy de acuerdo
14.	La mayoría de las personas con una enfermedad mental, no se esfuerzan lo suficiente para mejorarse.	**1** Muy en desacuerdo	**2** En desacuerdo	**3** Ni de acuerdo, ni en desacuerdo	**4** De acuerdo	**5** Muy de acuerdo
15.	Las personas con una enfermedad mental, rara vez representan un riesgo para la población.	**1** Muy en desacuerdo	**2** En desacuerdo	**3** Ni de acuerdo, ni en desacuerdo	**4** De acuerdo	**5** Muy de acuerdo

16.	El mejor tratamiento para la enfermedad mental son los medicamentos.	**1** **Muy en desacuerdo**	**2** **En desacuerdo**	**3** **Ni de acuerdo, ni en desacuerdo**	**4** **De acuerdo**	**5** **Muy de acuerdo**
17.	No me gustaría que una persona con una enfermedad mental trabajara con niños, incluso si su enfermedad estuviera bien compensada.	**1** **Muy en desacuerdo**	**2** **En desacuerdo**	**3** **Ni de acuerdo, ni en desacuerdo**	**4** **De acuerdo**	**5** **Muy de acuerdo**
18.	El personal de salud no necesita ser defensor de las personas que tienen una enfermedad mental.	**1** **Muy en desacuerdo**	**2** **En desacuerdo**	**3** **Ni de acuerdo, ni en desacuerdo**	**4** **De acuerdo**	**5** **Muy de acuerdo**
19.	No me importaría si una persona con una enfermedad mental fuera mi vecina/o.	**1** **Muy en desacuerdo**	**2** **En desacuerdo**	**3** **Ni de acuerdo, ni en desacuerdo**	**4** **De acuerdo**	**5** **Muy de acuerdo**
20.	Me cuesta sentir compasión por una persona que tiene una enfermedad mental.	**1** **Muy en desacuerdo**	**2** **En desacuerdo**	**3** **Ni de acuerdo, ni en desacuerdo**	**4** **De acuerdo**	**5** **Muy de acuerdo**

Por favor, continúe en la página siguiente.
