# Supplementary material for: Validation of the Opening Minds Scale and patterns of stigma in Chilean primary health care
Source: PLoS One. 2019 Sep 5;14(9):e0221825. doi: 10.1371/journal.pone.0221825 (PMC6728029; doi:10.1371/journal.pone.0221825)
Supplement: S2 File — Opening Minds Scale for Health Care Professionals (OMC-HC) in English. This is the original version of the Opening Minds Scale for Health Care Professionals (OMS-HC), which was cross-culturally adapted for use in the present study in Spanish (for Spanish version, see S1 File). (PDF) [file pone.0221825.s002.pdf]

A. Health Care Providers and Mental Illness[1].

The next few questions ask you to agree or disagree with a series of statements. There is no correct answer to each question. Please place an **"X"** in the box that best fits **your** opinion.

[1] Opening Minds, Mental Health Commission of Canada. Assessing Stigma Towards Mental Illness for Health Care Providers. Opening Minds Survey for Health Care Providers. Used with permission from Opening Minds, Mental Health Commission of Canada. Kassam A, Papish A, Modgill G, Patten S. The development and psychometric properties of a new scale to measure mental illness related stigma by healthcare providers: The Opening Minds Scale for Healthcare Providers (OMS-HC). BMC Psychiat 2012;12:62. Available at: http://bmcpsychiatry.biomedcentral.com/articles/10.1186/1471-244X-12-62.

		Strongly disagree	Disagree	Neither agree nor disagree	Agree	Strongly agree
**1.**	I am more comfortable helping a person who has a physical illness than I am helping a person who has a mental illness.					
**2.**	If a person with a mental illness complains of physical symptoms (e.g. nausea, back pain or headache), I would likely attribute this to their mental illness.					
**3.**	If a colleague with whom I work told me they had a managed mental illness, I would be as willing to work with him/her.					
**4.**	If I were under treatment for a mental illness I would not disclose this to any of my colleagues.					
**5.**	I would be more inclined to seek help for a mental illness if my treating healthcare provider was not associated with my workplace.					
**6.**	I would see myself as weak if I had a mental illness and could not fix it myself.					
**7.**	I would be reluctant to seek help if I had a mental illness.					
**8.**	Employers should hire a person with a managed mental illness if he/she is the best person for the job.					
**9.**	I would still go to a physician if I knew that the physician had been treated for a mental illness.					

		Strongly disagree	Disagree	Neither agree nor disagree	Agree	Strongly agree
**10.**	If I had a mental illness, I would tell my friends.					
**11.**	It is the responsibility of health care providers to inspire hope in people with mental illness.					
**12.**	Despite my professional beliefs, I have negative reactions towards people who have mental illness.					
**13.**	There is little I can do to help people with mental illness.					
**14.**	More than half of people with mental illness don't try hard enough to get better.					
**15.**	People with mental illness seldom pose a risk to the public.					
**16.**	The best treatment for mental illness is medication.					
**17.**	I would <u>not</u> want a person with a mental illness, even if it were appropriately managed, to work with children.					
**18.**	Healthcare providers do <u>not</u> need to be advocates for people with mental illness.					
**19.**	I would <u>not</u> mind if a person with a mental illness lived next door to me.					

**20.**	I struggle to feel compassion for a person with a mental illness.					
